# FNDC5 Promotes Adipogenic Differentiation of Primary Preadipocytes in Mashen Pigs

**DOI:** 10.3390/genes14010090

**Published:** 2022-12-28

**Authors:** Wei Hei, Ziwei You, Jiaqi An, Tianzhi Zhao, Jiao Li, Wanfeng Zhang, Meng Li, Yang Yang, Pengfei Gao, Guoqing Cao, Xiaohong Guo, Chunbo Cai, Bugao Li

**Affiliations:** 1 College of Animal Science, Shanxi Agricultural University, Jinzhong 030801, China; 2 Institute of Animal Sciences, Chinese Academy of Agricultural Sciences, Beijing 100193, China

**Keywords:** FNDC5, adipogenic differentiation, primary preadipocytes, Mashen pigs, ERK1/2

## Abstract

Fibronectin type III domain-containing protein 5 (FNDC5) plays an important role in fat deposition, which can be cut to form Irisin to promote fat thermogenesis, resulting in a decrease in fat content. However, the mechanism of FNDC5 related to fat deposition in pigs is still unclear. In this research, we studied the expression of FNDC5 on different adiposes and its function in the adipogenic differentiation of primary preadipocytes in Mashen pigs. The expression pattern of *FNDC5* was detected by qRT-PCR and Western blotting in Mashen pigs. *FNDC5* overexpression and interference vectors were constructed and transfected into porcine primary preadipocytes by lentivirus. Then, the expression of key adipogenic genes was detected by qRT-PCR and the content of lipid droplets was detected by Oil Red O staining. The results showed that the expression of *FNDC5* in abdominal fat was higher than that in back subcutaneous fat in Mashen pigs, whereas the expression in back subcutaneous fat of Mashen pigs was significantly higher than that of Large White pigs. In vitro, FNDC5 promoted the adipogenic differentiation of primary preadipocytes of Mashen pigs and upregulated the expression of genes related to adipogenesis, but did not activate the extracellular signal-regulated kinase (ERK) signaling pathway. This study can provide a theoretical basis for FNDC5 in adipogenic differentiation in pigs.

## 1. Introduction

Fat deposition is one of the important economic characters of pigs. Adipose tissue is mainly composed of mature adipocytes, which are distributed throughout the animal body and play a critical role in energy metabolism and homeostasis [1,2]. Although adipose tissue is significant for maintaining the energy metabolism of animals, excessive accumulation of fat will affect the carcass quality and reduce the value of pork [3]. In addition, there are many factors affecting the carcass quality of pigs, such as pig breed [4].

The Large Yorkshire pig, also known as the Large White pig, has a high lean rate and less fat deposition. The Mashen pig, a local breed in China, has the advantages of strong stress resistance, high fecundity, strong adaptability and high meat quality compared with the Large White pig [5]. However, it possesses a low feed conversion ratio, slow growth rate and more fat deposition. Adipogenesis in adult pigs is a highly regulated process that includes preadipocyte proliferation and subsequent differentiation into mature adipocytes [6]. However, limited research has been conducted on adipocyte differentiation in pigs.

Adipogenic differentiation requires precise regulation of genes. Fibronectin type III domain-containing protein 5 (FNDC5) was first discovered in 2002 and can be hydrolyzed by proteases to form Irisin, which performs its function by circulating throughout the body [7]. Previous research revealed that *FNDC5* is mainly expressed in skeletal muscle, but also in adipose, liver and cardiovascular tissues [8]. For myogenesis, existing research has demonstrated that FNDC5 can promote myogenic differentiation by activating the IL6 signaling pathway in C2C12 cells [9]. Similarly, FNDC5 can promote skeletal muscle regeneration by activating the proliferation and differentiation of skeletal muscle satellite cells after skeletal muscle injury [9]. In one study, the activities of oxidative metabolic enzymes and the expression of oxidative muscle fiber genes were increased by Irisin treatment in C2C12 myotubes [10]. The content of FNDC5 and Irisin decreased in skeletal muscle with hydrogen sulfide (H_2_S) deficiency, which can alter glucose metabolism [11]. Regarding adipose, FNDC5 can promote the browning of white adipose tissue [12]. Furthermore, the expression of *FNDC5* in visceral adipose and epididymal adipose of mice increased significantly after endurance training [13]. The expression of *FNDC5* in subcutaneous adipose tissue of obese patients was significantly higher than that of normal weight people [14]. Nevertheless, the specific function of FNDC5 during the adipogenic differentiation of preadipocytes in pigs remains poorly understood.

The extracellular signal-regulated kinase1/2 (ERK1/2) signaling pathway plays an important role in regulating adipocyte differentiation. Previous research revealed that the specific inhibitor U0126 of ERK1/2 can significantly inhibit adipogenic differentiation in 3T3-L1 cells [15]. The fat of *ERK1*^−/−^ mice reduced, and the insulin sensitivity enhanced [16]. Furthermore, the expression of CCAAT/enhancer binding protein alpha (C/EBPα), peroxisome proliferator-activated receptor gamma (PPARγ), and fatty-acid-binding protein 4 (FABP4) decreased significantly during the adipogenic differentiation in mouse mesenchymal stem cells with ERK1/2 inhibitor addition, and the lipid accumulation decreased significantly [17].

In the present study, we focused on the difference in fat deposition between Mashen pigs and Large White pigs. FNDC5 caught our attention, because it is expressed differentially in subcutaneous adipose of the two breeds. This study explored the regulatory effect of FNDC5 during the adipogenic differentiation of preadipocytes in pigs.

## 2. Materials and Methods

### 2.1. Ethics Statement

All procedures performed on animals were approved by the Shanxi Agricultural University Animal Care and Ethical Committee, China (approval no. SXAU-EAW-P002003).

### 2.2. Sample Preparation

Under the same feeding and management conditions, 1-, 90- and 180-day-old Mashen pigs (castrated males, *n* = 12; females, *n* = 12) and 90-day-old Large White pigs (castrated males, *n* = 4; females, *n* = 4) were selected from Datong Pig Farm (Datong, Shanxi, China). All the tissue samples from Mashen pigs and Large White pigs were collected strictly according to the anatomy of the pigs. The Mashen pig was used for the isolation of porcine preadipocytes based on previously established methods [18]. Porcine preadipocytes were isolated and cultured from subcutaneous adipose tissue of Mashen pigs at 7 days of age. The tissues were dissected and digested with collagenase II (2 mg/mL, Gibco, Carlsbad, CA, USA, Cat. 17101015) for 1 h. After digestion was terminated with Dulbecco’s Modified Eagle Medium (DMEM) (Gibco, Carlsbad, CA, USA, Cat. 11965118) including 10% fetal bovine serum (FBS) (Gibco, Carlsbad, CA, USA, Cat. 10099141), the mixture was centrifuged at 1200 rpm for 15 min, and then the supernatant was discarded and the cells were re-suspended. After filtration, the filtrate was inoculated in a 60 mm Petri dish. The obtained cells were cultured in low-glucose DMEM containing 10% FBS and 1% penicillin streptomycin (Gibco, Carlsbad, CA, USA, Cat. 15140122) in a 5% CO_2_ incubator at 37 °C. The culture medium was changed every 2 days.

### 2.3. Adipogenic Induction and Oil Red O Staining

When the density of porcine preadipocytes reached 90%, they were digested with trypsin and inoculated into 6-well plates. The culture medium was changed after 2 days. When cells reached 100% confluence, they were induced to adipogenic differentiation with differentiation medium, containing low-glucose DMEM, bovine insulin (5 mg/mL, Solarbio, Beijing, China, Cat. I8040), 3-isobutyl-1-methylxanthine (0.5 mM/L, Solarbio, Beijing, China, Cat. I8450), dexamethasone (1 μM/L, Solarbio, Beijing, China, Cat. D8040), indomethacin (0.5 mM/L, Sigma-Aldrich, Billerica, MA, USA, Cat. I7378), and 10% FBS. After 2 days of differentiation, the medium was replaced with maintenance medium containing low-glucose DMEM and 5 μg/mL bovine insulin. The medium was refreshed every 2 days and the morphological changes during the adipogenic differentiation were observed under a microscope magnified 100 times (Leica, Wetzlar, Germany).

To stain the lipids, the cells were washed with ice-cold PBS (Solarbio, Beijing, China, Cat. P1010) and fixed with 4% paraformaldehyde at 4 °C for 30 min. After that, the cells were rinsed with 60% isopropyl alcohol for 1 min and incubated with Oil Red O working solution (Solarbio, Beijing, China, Cat. O8010) for 1 h. After the cells were washed several times with double-distilled water, the images were captured using a microscope magnified 100 times.

### 2.4. RNA Extraction and cDNA Synthesis

Total RNA was extracted using RNAiso Plus (Takara, Shiga, Japan, Cat. 9108) according to the manufacturer′s protocol. The RNA concentration and integrity were determined using an ND-2000 spectrophotometer (NanoDrop Technologies, DE) and 1% agarose gel electrophoresis, respectively. Thereafter, total RNA (500 ng) was converted into complementary DNA (cDNA) using a PrimeScript RT reagent Kit with gDNA Eraser (Takara, Shiga, Japan, Cat. RR047A) under the following conditions: 37 °C for 15 min and 85 °C for 15 s.

### 2.5. Quantitative Real-Time PCR (qRT-PCR)

Quantitative real-time PCR was performed using TB Green Premix Ex Taq II (Takara, Shiga, Japan, Cat. RR820A) on an ABI-7500 (Life Technologies) under the following conditions: pre-denaturation at 95 °C for 30 s; 45 cycles of 95 °C for 5 s and 60 °C for 34 s; one cycle of 95 °C for 15 s and 60 °C for 1 min; 95 °C for 30 s. The expressions of all genes were normalized to *18S* rRNA. The 2^−ΔΔCt^ formula was used to estimate relative expression levels. Primer sequences used for qRT-PCR analyses were listed in Table 1.

### 2.6. Paraffin Section and HE Staining

The adipose tissues were immersed with 4% paraformaldehyde for 24 h, and dehydrated with gradient alcohol. The tissue was hyalinized with xylene, then immersed in wax to be embedded and trimmed. The repaired wax block was placed into the slicer, and then picked up with slides after spreading. After drying, HE staining was carried out. The slices were placed in xylene and alcohol for dewaxing, then washed with PBS 3 times and stained in hematoxylin dye for 5 min. The excess dye was washed with PBS, and the slices were placed in gradient alcohol and eosin dye for 5 min. Finally, the slices were dehydrated and sealed with neutral resin glue.

### 2.7. Western Blotting

To extract total proteins from cells, they were treated with a lysis buffer supplemented with protease inhibitors (Solarbio, Beijing, China, Cat. P6730), phosphatase inhibitors (Solarbio, Beijing, China, Cat. P1260) and phenylmethylsulfonyl fluoride (PMSF) (Solarbio, Beijing, China, Cat. P8340). The extracted proteins were loaded on 10% SDS-polyacrylamide gels and transferred onto nitrocellulose filter membranes (Solarbio, Beijing, China, Cat. HATF00010). Then, the membrane was rinsed with PBS and sealed with 5% skim milk in PBS for 1 h. Western blotting was performed using the standard method for the following proteins with corresponding detection antibodies (in brackets): FNDC5 (1:1000, Abcam, Cambridge, UK, Cat. ab174833), anti-ERK1/2 and anti-phospho-ERK1/2 (1:2000, Cell Signaling Technology, Boston, MA, USA, Cat. 4370S). β-actin (1:2500, Proteintech, Wuhan, China, Cat. 205361AP) was used as an internal reference. After being washed with TBST, the membranes were incubated with a secondary antibody (1:10000, LI-COR, Lincoln, NE, USA, Cat. 92632211) for one hour at room temperature. Then, the membranes were washed with TBST again and imaged with the Odyssey CLX imaging system (LI-COR, USA).

### 2.8. Lentiviral-Mediated Transfection

The pair of short hairpin oligonucleotides (GCGATGCACAACTTTGCAAGT) targeting the open reading frame (ORF) of *FNDC5* was designed and synthesized by GenePharma. Both vector construction (OE-*FNDC5* and sh-*FNDC5*) and lentivirus package were also commissioned by GenePharma. The preadipocytes were inoculated into 12-well plates. When the cell density was about 50%, the appropriate amount of lentivirus was directly added to the medium for infection. The medium was changed after 24 h.

### 2.9. Statistical Analysis

The two groups of samples were compared by the Student’s *t*-test, where *p* < 0.05 (*) and *p* < 0.01 (**) indicate statistically significant differences. Comparisons between three or more samples were performed using one-way analysis of variance (ANOVA), and Duncan’s method was used for multiple comparisons. GraphPad Prism (Version 8, San Diego, CA, USA) was used to conduct the statistical analysis and plotting.

## 3. Results

### 3.1. The Expression Profile of FNDC5 in Mashen Pigs

**Figure 1:**
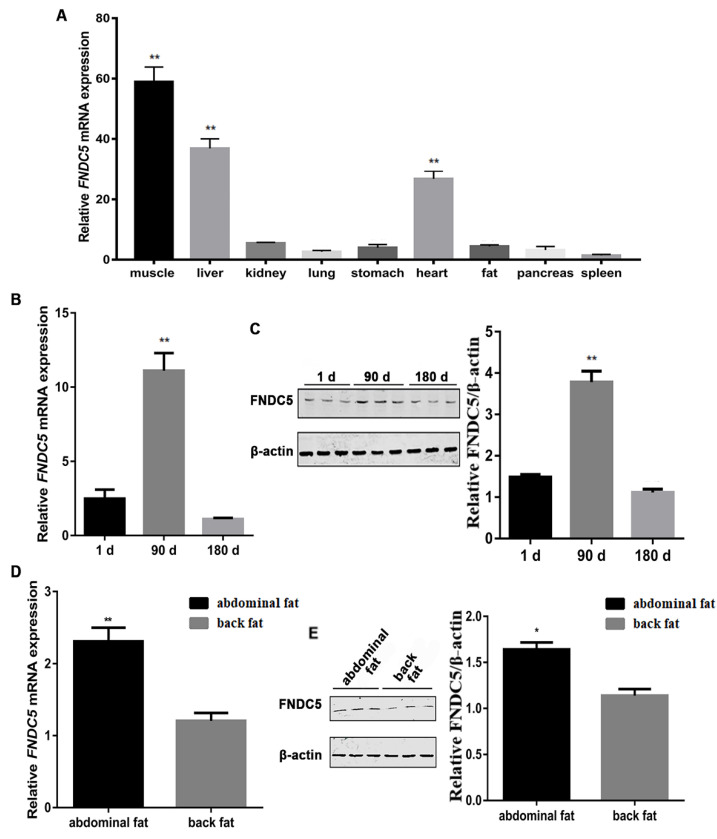
The expression profile of *FNDC5* in Mashen pigs. (**A**) The expression of *FNDC5* in different tissues of 90-day-old Mashen pigs. (**B**,**C**) The *FNDC5* expression in back fat at 1, 90 and 180 days old by qRT-PCR and Western blotting. (**D**,**E**) The *FNDC5* expression in abdominal and back fat in 90-day-old Mashen pigs. Notes: “**” means extremely significant difference (*p* < 0.01) and “*” means significant difference (*p* < 0.05).

The expression of *FNDC5* was higher in muscle, liver and heart, but lower in fat in 90-day-old Mashen pigs (Figure 1A). The *FNDC5* expression in back fat was higher at 90 days old than that at 1 and 180 days old by qRT-PCR (Figure 1B) and Western blotting (Figure 1C) (*p* < 0.01). Furthermore, the qRT-PCR (Figure 1D) and Western blotting (Figure 1E) demonstrated that the *FNDC5* expression in abdominal fat of Mashen pigs was significantly higher than that in back fat (*p* < 0.01).

### 3.2. Difference of Back Fat between Mashen and Large White Pigs

**Figure 2:**
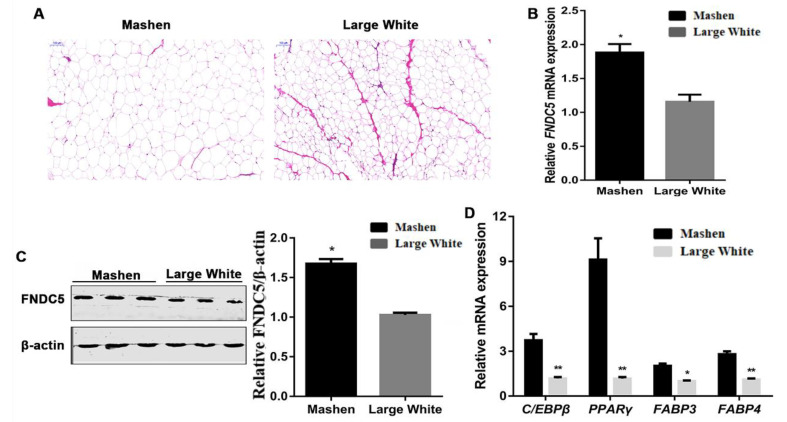
Difference in back fat between Mashen and Large White pigs. (**A**) HE staining results. (**B**) The expression of *FNDC5*. (**C**) Western blotting result of FNDC5. (**D**) The expression of adipogenic marker genes. Scale bars: 100 μm. Notes: “**” means extremely significant difference (*p* < 0.01) and “*” means significant difference (*p* < 0.05).

The fat area in the back fat tissue of Mashen pigs was obviously larger than that in Large White pigs (Figure 2A). The *FNDC5* expression of back fat in Mashen pigs was significantly higher than that in Large White pigs (*p* < 0.05) (Figure 2B), and the result of Western blotting was similar (*p* < 0.05) (Figure 2C). Moreover, qRT-PCR was performed to determine the expression of the adipogenic marker genes (Figure 2D). Compared with Large White pigs, the expressions of *CEBP/β* (*p* < 0.01), *PPARγ* (*p* < 0.01), *FABP3* (*p* < 0.05) and *FABP4* (*p* < 0.01) in Mashen pigs were significantly increased.

### 3.3. FNDC5 Promoted the Adipogenic Differentiation of Porcine Preadipocytes in Mashen Pigs

**Figure 3:**
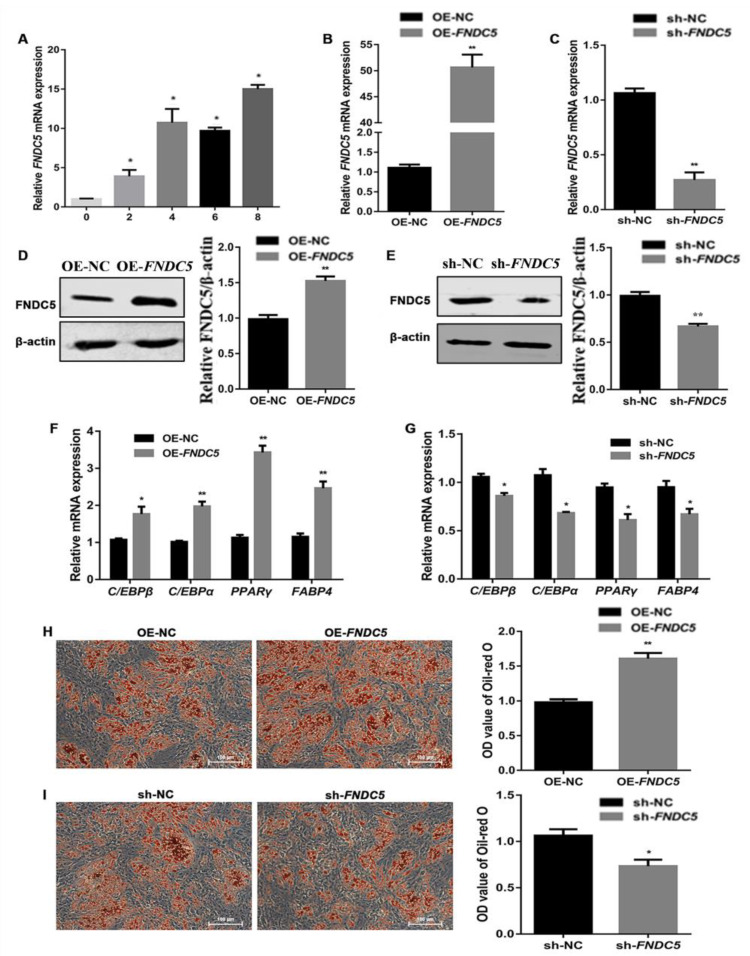
Regulation of FNDC5 in adipogenic differentiation in porcine preadipocytes. (**A**) The expression of *FNDC5* at 0, 2, 4, 6 and 8 days during adipogenic differentiation in porcine preadipocytes. (**B**,**C**) The expression of *FNDC5* after transfecting the *FNDC5* overexpression and interference vectors. (**D**,**E**) Western blotting results of FNDC5 after transfecting the *FNDC5* overexpression and interference vectors. (**F**,**G**) The expression of adipogenic marker genes after transfecting the *FNDC5* overexpression and interference vectors. (**H**,**I**) The Oil Red O staining and OD value reflecting the content of triglyceride after transfecting the *FNDC5* overexpression and interference vectors. OE-NC and sh-NC mean transfecting the negative control overexpression and interference vectors, respectively. OE-*FNDC5* and sh-*FNDC5* mean transfecting the *FNDC5* overexpression and interference vectors, respectively. The same as below. Scale bars: 100 μm. Notes: “**” means extremely significant difference (*p* < 0.01) and “*” means significant difference (*p* < 0.05).

The expression of *FNDC5* was detected during the adipogenic differentiation stage of porcine preadipocytes. The results indicate that the expression of *FNDC5* increased gradually over the adipogenic differentiation time (Figure 3A). To further confirm the function of FNDC5 during the adipogenic differentiation of porcine preadipocytes, the overexpression and interference vectors of *FNDC5* were transfected into porcine preadipocytes by lentivirus. The expression of *FNDC5* was significantly increased and decreased after transfecting the overexpression and interference vectors, respectively (Figure 3B,C). Western blotting showed similar results (Figure 3D,E). After the overexpression and interference of *FNDC5* in porcine preadipocytes, the expression of adipogenic marker genes was detected on the fourth day of adipogenic differentiation. The expression of *C/EBPβ*, *C/EBPα*, *PPARγ* and *FABP4* was significantly upregulated by *FNDC5* overexpression (Figure 3F) and downregulated by *FNDC5* interference (Figure 3G). Furthermore, Oil Red O staining on the 8th day of adipogenic differentiation showed a significant increase in lipid droplet formation upon *FNDC5* overexpression (Figure 3H). In contrast, *FNDC5* interference reduced lipid accumulation (Figure 3I).

### 3.4. FNDC5 Had No Effect on ERK1/2 Phosphorylation during Adipogenic Differentiation in Porcine Preadipocytes

**Figure 4:**
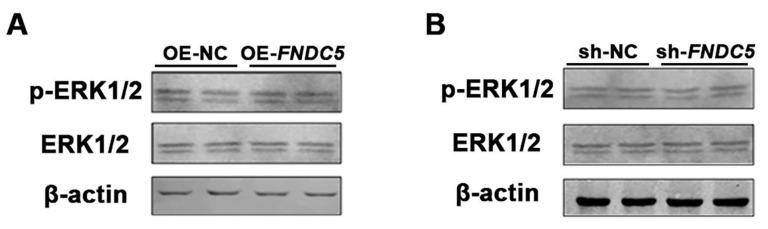
Effect of FNDC5 on phosphorylation of ERK1/2. (**A**,**B**) Western blotting of ERK1/2 and phosphorylated ERK1/2 after transfecting the FNDC5 overexpression and interference vectors in porcine preadipocytes. β-actin was used as the internal reference protein.

Western blotting of ERK1/2 and phosphorylated ERK1/2 was performed on the 8th day of adipogenic differentiation after transfecting the FNDC5 overexpression and interference vectors in porcine preadipocytes. There was no significant difference in P-ERK1/2 content after overexpressing and interfering *FNDC5* in porcine preadipocytes (Figure 4A,B).

## 4. Discussion

*FNDC5* is mainly expressed in skeletal muscle and less in adipose tissue [19,20]. However, FNDC5 plays an important role in regulating body fat deposition and cell adipogenic differentiation. Irisin can promote the differentiation of mesenchymal stem cells (MSCs) into beige adipocytes [21]. Moreover, Perez-Sotelo et al. reported that the expression level of *FNDC5* in subcutaneous adipose tissue of obese patients was significantly higher than that of normal weight subjects [14]. Furthermore, *FNDC5* upregulated the expression of genes related to lipolysis and promoted browning in 3T3-L1 adipocytes [22]. In our study, we found that the expression of *FNDC5* in back subcutaneous fat of Mashen pigs was significantly higher than that of Large White pigs. Presumably, this is because Mashen pigs have a stronger ability to deposit fat compared to Large White pigs. Meanwhile, our study suggested that the expression of *FNDC5* in abdominal fat was higher than that in back subcutaneous fat in Mashen pigs. These findings suggest that FNDC5 plays an important role in adipogenesis of Mashen pigs.

At present, there are inconsistent reports on the regulation of FNDC5 during the differentiation of preadipocytes. Huh et al. found that FNDC5 inhibited the adipogenic differentiation of human adipocytes by downregulating the expression of fatty acid synthase (*FAS*) [23]. In addition, interference with FNDC5 promoted adipogenic differentiation of C3H10T1/2 cells, accompanied by a pronounced increase in lipid droplet content [14]. On the contrary, Dong et al. reported that the lipid content was visibly increased after overexpressing *FNDC5* in goat preadipocytes [24]. Therefore, the function of FNDC5 during the differentiation of porcine preadipocytes requires more in-depth research. This study found that overexpressing *FNDC5* in porcine preadipocytes resulted in upregulated expression of adipogenic marker genes and increased lipid accumulation. In brief, FNDC5 promoted the differentiation of porcine preadipocytes. It is universally accepted that FNDC5 can be cleaved to form irisin protein, which is secreted into blood circulation and then enters the adipose tissues [25]. Irisin can alleviate adipogenesis and increase energy consumption [26]. Consistently, overexpression of Irisin in 3T3-L1 cells downregulated the expression of *PPARγ* and *FABP4*, and significantly reduced lipid droplet content [27]. Li et al. also demonstrated that the addition of Irisin recombinant protein to human preadipocytes predominantly attenuates the lipid accumulation [28]. Therefore, it is a controversial issue regarding the regulatory roles of FNDC5 and Irisin in adipogenic differentiation, and further investigation is required to clarify this issue in the future.

The ERK1/2 signaling pathway was shown to carry out a key role in regulating porcine adipogenesis. It was observed that progranulin inhibits adipogenesis in porcine preadipocytes partially through ERK1/2 activation-mediated PPARc phosphorylation [29]. Moreover, vascular endothelial growth factor receptor type 2 (VEGFR2) can activate the ERK pathway to induce the differentiation of adipose-derived mesenchymal stem cells (AMSCs) to endothelial cells (ECs) [30]. Sun et al. found that platelet-derived growth factor receptor α (PDGFRα) promoted adipogenesis in porcine intramuscular preadipocytes through activating the ERK1/2 signaling pathway [31]. Various reports found that FNDC5 can regulate many biological functions through the ERK signaling pathway. Exogenous addition of Irisin recombinant protein can enhance endothelial cell proliferation through regulating the ERK1/2 signaling pathway and improve cell angiogenesis [32]. In addition, FNDC5 can stimulate transient activation of ERK1/2 in Alzheimer’s disease mouse models [33]. Previous studies found that FNDC5 can promote C2C12 cell proliferation via the ERK1/2 signaling pathway [34]. However, there is no report that FNDC5 regulates the differentiation of porcine preadipocytes by the ERK1/2 signaling pathway. Our results showed that FNDC5 had no significant effect on the ERK1/2 signaling pathway in adipogenic differentiation of porcine preadipocytes. Based on this result, we deem that FNDC5 regulates the differentiation of porcine preadipocytes by other signaling pathways. For example, overexpression of *FNDC5* can activate the p38 MAPK pathway in 3T3-L1 adipocytes [35]. Additionally, the mTORC1 signaling pathway was activated and fat content was significantly increased in *FNDC5* knockout mice [36]. In addition, FNDC5 has been reported to improve insulin sensitivity in mice by activating the AMPK signaling pathway [37].

In conclusion, our study suggests that FNDC5 promotes the differentiation of porcine adipocytes, which may be one of the causes of the difference in fat deposition between Mashen pigs and Large White pigs. Furthermore, FNDC5 has no significant effect on the ERK1/2 signaling pathway in porcine preadipocytes. These results provide a theoretical basis for the regulation of FNDC5 in adipose development.

## Figures and Tables

**Table 1:** Primer information.

Genes	Primer Sequence (5’-3’)	GenBank No.
*FNDC5*	F: TGCAGGCCATCTCCATTCAG	XM_021095830.1
	R: ATATTGGCGGCAGAAGAGGG	
*FABP3*	F: TGACACTGGATGGAGGCAAA	NM_001099931.1
	R: TGGGTGAGTGTCAGGATGAGT	
*PPARγ*	F: CTATTCCATGCTGTCATGGGTG	NM_214379.1
	R: ACCATGGTCACCTCTTGTGA	
*FABP4*	F: TGGTACAGGTGCAGAAGTGG	NM_001002817.1
	R: TTCTGGTAGCCGTGACACCT	
*C/EBPα*	F: AGAACAGCAACGAGTACCGG	NM_001123135.1
	R: GCTCCAGCACCTTCTGTTGA	
*C/EBPβ*	F: CTGGAGACGCAGCATAAGGT	NM_001199889.1
	R: TGCTTGAACAAGTTCCGCAG	
*18S*	F: ATGCCAGAGTCTCGTTCGTTAT	NR_046261.1
	R: CGGACAGGATTGACAGATTGAT	

F: forward; R: reverse.

## Data Availability

All data generated or analyzed during this study are included.
